# Magnetic Particle Imaging meets Computed Tomography: first simultaneous imaging

**DOI:** 10.1038/s41598-019-48960-1

**Published:** 2019-09-02

**Authors:** Patrick Vogel, Jonathan Markert, Martin A. Rückert, Stefan Herz, Benedikt Keßler, Kilian Dremel, Daniel Althoff, Matthias Weber, Thorsten M. Buzug, Thorsten A. Bley, Walter H. Kullmann, Randolf Hanke, Simon Zabler, Volker C. Behr

**Affiliations:** 1 Department of Experimental Physics 5 (Biophysics), University of Würzburg, 97074 Würzburg, Germany; 2 Department of Experimental Physics (X-Ray Microscopy), University of Würzburg, 97074 Würzburg, Germany; 3 Institute of Medical Engineering, University of Applied Sciences Würzburg-Schweinfurt, 97421 Schweinfurt, Germany; 4 Department of Diagnostic and Interventional Radiology, University Hospital Würzburg, 97080 Würzburg, Germany; 5 Institute of Medical Engineering, University of Lübeck, 23562 Lübeck, Germany; 6 Fraunhofer Development Center X-ray Technology EZRT, 97074 Würzburg, Germany; 7 Present Address: Magnetic Insight Inc., Alameda, CA USA

**Keywords:** Biomedical engineering, Applied physics, Imaging techniques

## Abstract

Magnetic Particle Imaging (MPI) is a promising new tomographic modality for fast as well as three-dimensional visualization of magnetic material. For anatomical or structural information an additional imaging modality such as computed tomography (CT) is required. In this paper, the first hybrid MPI-CT scanner for multimodal imaging providing simultaneous data acquisition is presented.

## Introduction

Magnetic Particle Imaging (MPI) is a tomographic method that detects the three-dimensional distribution of superparamagnetic iron-oxide nanoparticles (SPIONs)^1^. MPI signal is solely obtained from particles, but not from surrounding tissue. Thus, combining MPI with an additional tomographic modality, allows for registering the particle signal and the anatomical information, which enhances the diagnostic potential^2^. Since MPI is based on the nonlinear response of SPIONs regarding time-varying magnetic fields, a combination with another imaging modality utilizing magnetic fields - such as magnetic resonance imaging (MRI) - is obvious. In previous experiments the feasibility of MPI-MRI hybrid scanners has been shown. Initially, a low-field MRI machine (30 mT/1.1 mT) was integrated with a Traveling Wave MPI device^3–5^ and later a regular-field MRI machine (500 mT) was combined with a classical MPI device^6^. In both cases the much higher hardware demands and longer acquisition times of MRI – in contrast to MPI systems (several minutes vs. several milliseconds^7^) – put both modalities at a disadvantage.

The combination of MPI with computed tomography (CT) overcomes the timing issue and was firstly presented as fusion MPI-CT imaging, whereby both images were acquired successively^8–10^, which is not appropriate for simultaneous MPI-CT imaging. A hybrid MPI-CT device implies certain challenges such as the usage of X-rays for imaging, which requires a ‘free’ view through the sample. Conventional MPI scanners are based on a closed-bore design providing an efficient generation of magnetic field gradients^11^. However, an MPI-CT hybrid system requires an open MPI concept. Several single-sided field-free point (FFP) as well as field-free line (FFL) MPI approaches have been presented in the past, which demonstrate the general feasibility^12–14^ though it requires much more effort to reach the performance of a closed MPI design with a single sided MPI design.

In 2008 an MPI scanner concept was presented using a field free line (FFL) instead of an FFP for encoding the volume of interest^15^. A common feature between CT and FFL MPI is the acquisition scheme: Both technologies acquire projections. However, most FFL concepts are based on complex hardware designs for either electrical or mechanical rotation of the FFL or the sample^16,17^. Typically, a direct view through the system is prevented by the electromagnetic components.

Recently, a concept for generating a static FFL utilizing Halbach rings^18,19^ was presented by Weber *et al*.^20^. The Halbach rings are integrated in a rotating gantry. Although, the initial concept features a closed-bore design to generate a magnetic field with a high gradient, this approach can easily be redesigned to offer an open design to provide a ‘window’ for CT imaging.

Reducing the number of permanent magnets per Halbach ring reduces they magnetic field gradient but provides more open space required for CT. In this manuscript, the concept of the MPI encoding scheme utilizing two Halbach rings is presented followed by the assembly of the first MPI-CT hybrid scanner.

## Material and Methods

A novel MPI-CT hybrid scanner is proposed in the following section. An open micro-CT system is utilized providing easy access to all parts and areas offering sufficient space for MPI hardware integration.

### Halbach Rings for Static FFL Generation

Halbach arrays were introduced by K. Halbach in 1980 and describe an arrangement of permanent magnets increasing the magnetic flux on one side, while reducing it on the other side^18^. Halbach cylinders or rings are based on *N* permanent magnets which are spatially rotated around a symmetry axis where the angle Δ*φ* between the orientation vectors of adjacent magnets is chosen to fulfill the following equation^19^:

1 $$N\cdot {\rm{\Delta }}\phi =(k+1)\cdot 360^\circ ,\,k\in {{\mathbb{N}}}_{0}$$

**Figure 1:**
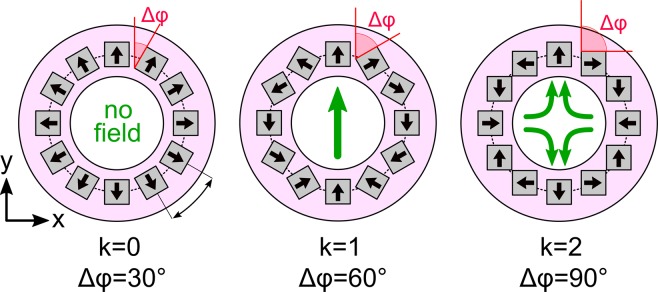
Halbach rings with different angles Δφ between adjacent permanent magnets and the resulting magnetic field in the center of the Halbach rings. The black arrows indicate the magnetization of the permanent magnets, the green arrows the resulting magnetic field lines generated by the Halbach rings.

By increasing parameter *k*, which increases Δ*φ*, different magnetic field profiles can be designed as indicated in Fig. 1.

**Figure 2:**
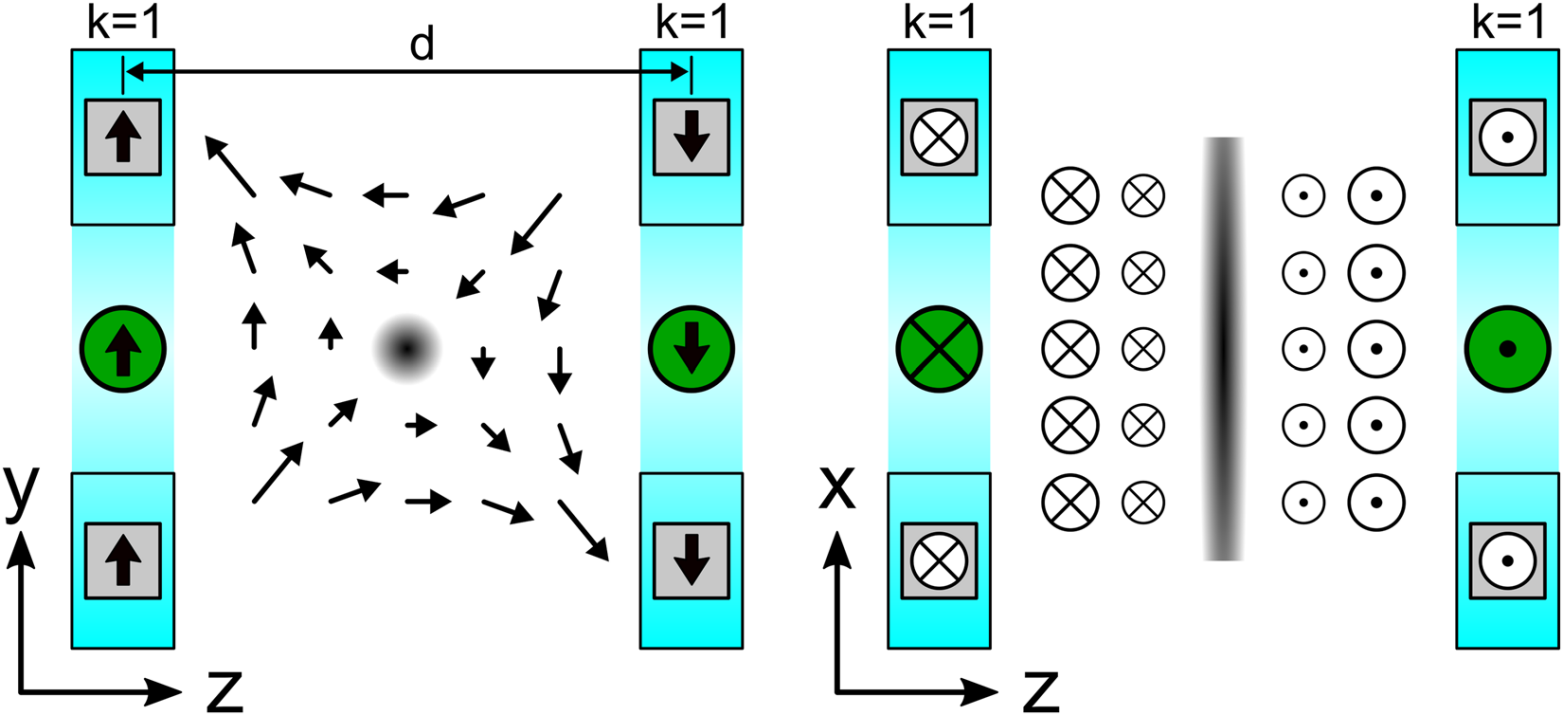
Generation of static FFL in the x-z plane by using two anti-parallel Halbach rings with k = 1 configuration. The dark regions in the center of both figures (dot on the left and line on the right) indicate the field free line. The bold black arrows indicate the magnetization of the permanent magnets. The green filled circles indicate the direction of the magnetic field for each Halbach ring in their center. The magnetic field profile is indicated by a vector field plot.

Halbach rings featuring *k* = 1 generate a homogeneous magnetic field, which is orientated in the plane of the ring. To generate a static FFL, two such Halbach rings can be utilized. Both rings face each other with opposing magnetization direction as depicted in Fig. 2. This results in a static FFL with a gradient strength *G*_z_ located in the center of both Halbach rings (see Fig. 2) and orientated along *x*-axis (*y*/*z* plane) providing a ‘window’ for acquiring CT projections.

### Halbach based MPI FFL Scanner Design

**Figure 3:**
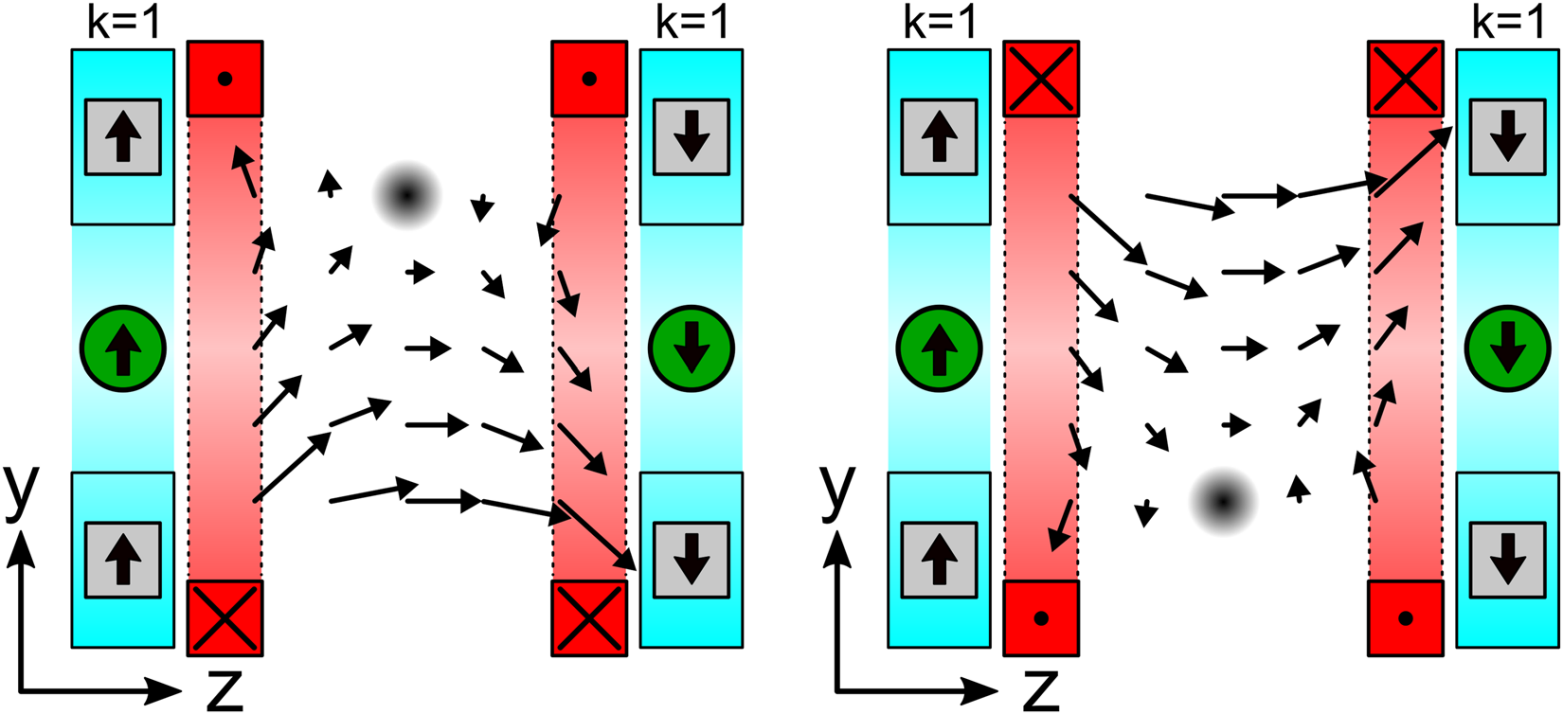
A varying magnetic field orientated along the z-axis shifts the FFL (x-direction) up and down along the y-direction. To provide the CT ‘window’ the magnetic field is generated by two separated solenoid coils (red). The arrows indicate the magnetic field directions.

For imaging a 2D projection, the FFL must be shifted along the y-direction. This is performed with an additional magnetic field orientated in *z*-direction generated by two separated solenoid coils assembled as shown in Fig. 3.

The required magnetic field strength to shift the FFL to cover the entire FOV depends on the gradient strength *G*_*z*_. The following equation describes the magnetic field inside the Halbach rings:

2 $$\overrightarrow{H}(\overrightarrow{x})={\textstyle (}\begin{array}{ccc}0 & 0 & 0\\ 0 & 1 & 0\\ 0 & 0 & -1\end{array}{\textstyle )}{G}_{z}\overrightarrow{x}$$

Equation 2 and the size of the FOV *d*_FOV_ can be used to determine the required magnetic field strength *H*_y_:

3 $${H}_{y}={G}_{z}\cdot {d}_{FOV}/2$$

### Halbach Ring Assembly

**Figure 4:**
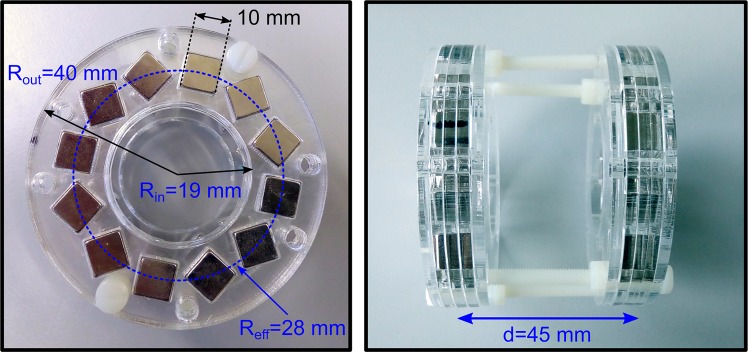
Halbach ring assembled in k = 1 configuration. The magnetic field strength in the center of each ring is 80 mT. The gradient strength in the center of both rings is about 4.3T/m at a center-to-center distance d = 45 mm.

The Halbach rings were assembled from 12 cubic neodymium magnets (NIB-N-52) with a side length of 10 mm (see Fig. 1). They are arranged in a *k* = 1 configuration in an acrylic glass holder (inner diameter *R*_in_ = 19 mm, outer diameter *R*_out_ = 40 mm) on an effective radius *R*_eff_ = 28 mm (see Fig. 4). Each Halbach ring generates a homogeneous magnetic field of about 80 mT in their common center (measured with a Hall-sensor). The center-to-center distance *d* between the two rings is 45 mm generating a magnetic field gradient of about 4.3 T/m in the FFL region.

### MPI FFL Scanner Assembly

**Figure 5:**
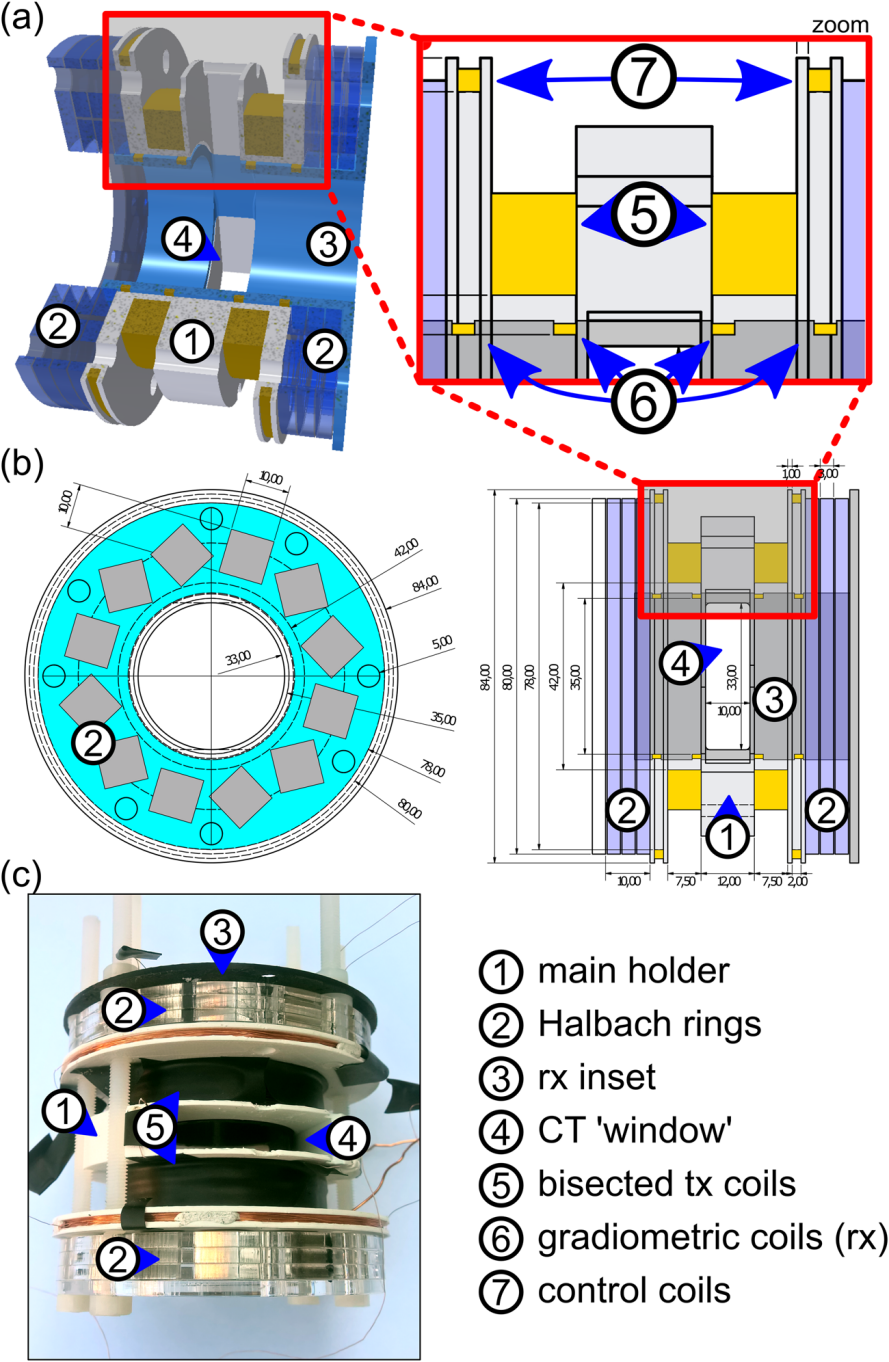
(**a**) Three-dimensional sketch of the scanner setup. (**b**) CAD drawing of the MPI FFL setup: left: front view, right: side view and zoomed view. Both Halbach rings enclose the main holder consisting the bisected excitation coil. The four-sectional gradiometric receive coil is assembled on the RX inset fitting in the main holder. The CT ‘window’ is set to 33 × 10 mm². The inner bore diameter is 33 mm. (**c**) Image of the fully assembled MPI parts of the MPI-CT hybrid scanner.

For shifting the FFL along the *y*-direction and providing the CT ‘window’ (Fig. 5 #4), a bisected solenoid (transmit coil – tx – Fig. 5 #5) is wound on the 3D-printed main holder (material: PLA, printer: N2, Raise3D, USA) (Fig. 5 #1). For each section enameled copper wire (diameter 0.6 mm) is wound in 130 loops resulting in a resistance of 2.2 Ω (DC) and inductance of 2.45 mH. The bisected excitation coil is driven at a frequency of 10250 Hz (series resonant circuit) with an audio amplifier (t.amp TS2400MK-X, Thomann, Germany) generating a magnetic field amplitude of ±100 mT in the center of the setup. This is sufficient for shifting the FFL through the FOV. For signal acquisition a four-sectional gradiometric receive coil (rx – Fig. 5 #6)^21^ is assembled on a rx inset (Fig. 5 #3) fitting in the main holder (enameled copper wire with of 250 µm diameter, 50 windings per section resulting in a resistance of 30 Ω (DC)). Additional coils are used for calibration and setup control (see Fig. 5 #7).

### Micro CT Scanner

**Figure 6:**
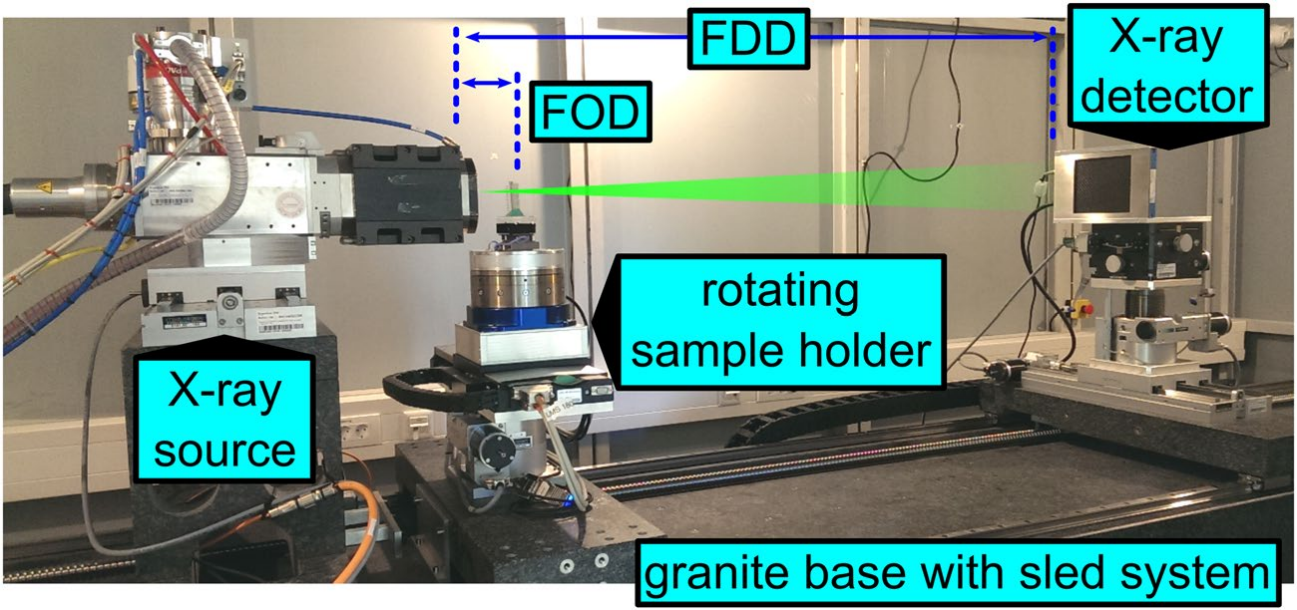
Fully assembled micro-CT “MetRIC” setup inside the shielded cabin.

The Micro-CT system “MetRIC” is an inhouse scanner at Fraunhofer EZRT Würzburg. X-ray source and detector are set up on a 4.4 m long granite base with the sample manipulator (two linear axes, one rotational axes) in between. The source is an open transmission anode with a tungsten target. For detection, a standard flat panel device is used. The geometric magnification of the object in MetRIC is variable and defined by the focus-detector distance (FDD) as well as by the focus-object distance (FOD) as *M* = *FDD/FOD*. Thus, the effective voxel sampling (isotropic size of a voxel) during CT is *∆v* = *∆x*/*M* = 19.4 µm with Δ*x* = 74.8 µm being the detector pixel size. Figure 6 shows a side view of the scanner inside the radiation shielded cabin.

### Hybrid MPI-CT Scanner

**Figure 7:**
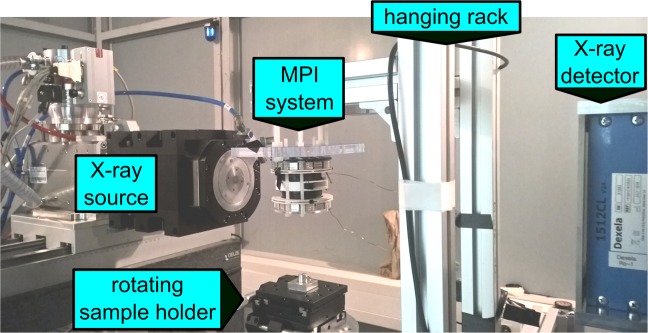
Image of the MPI-CT system: left the X-ray source, in the center the MPI scanner and on the bottom the rotating sample holder.

All parts of the hybrid scanner (described in Sec. II.2 and II.3.) are assembled on a sled-system providing a free adjustment of distances between X-ray source, sample holder and X-ray detector offering a flexible access to all parts (see Fig. 6). The MPI system is assembled on a sled holding a hanging rack providing a direct access to the common FOV from below (see Fig. 7).

### Bi-modal Samples

**Figure 8:**
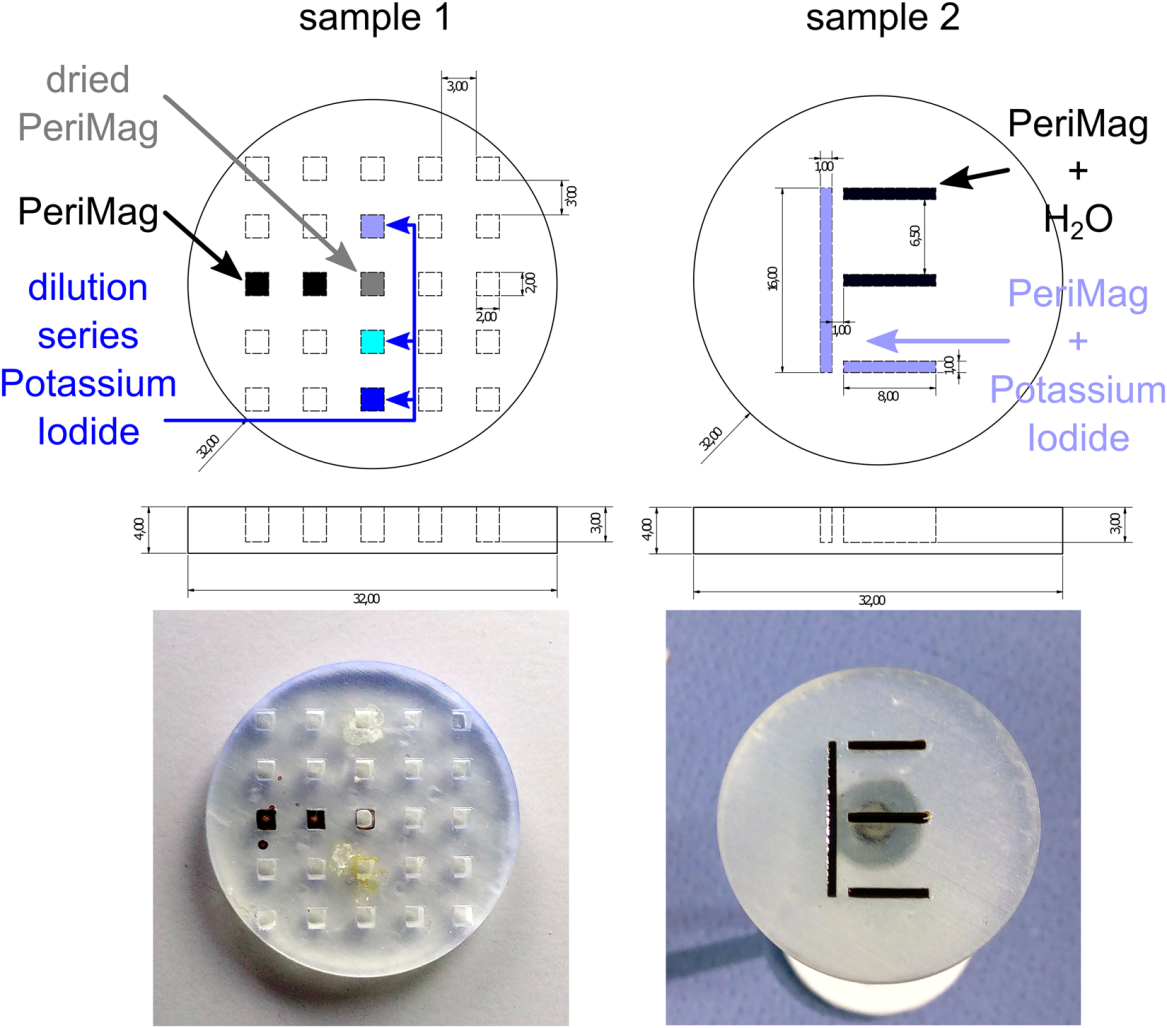
CAD sketch of the samples: **Left**: point sample (sample 1) filled with undiluted PeriMag, dried PeriMag and a dilution series of potassium iodide (KI). **Right**: letter sample (sample 2) is partially filled with mixtures of PeriMag and KI forming the letter “L” and PeriMag and H_2_O completing the letter “E”.

Different test samples were constructed and 3D-printed (see Fig. 8) (material: clear resin, printer: Form2, formlabs, USA). For MPI-CT hybrid imaging, the samples were prepared with PeriMag (conc. 56 mg Fe/ml, Micromod, Germany) contributing to the MPI signal and potassium iodide (KI) establishing CT visibility. Several dots of sample 1 (see Fig. 8) were filled with PeriMag, dried PeriMag and a dilution series of KI (conc. 267 mg/ml, 94 mg/ml, 16 mg/ml). The parts of the letter in sample 2 (see Fig. 8) were filled with diluted PeriMag (1:1 with H2O) as well as an 1:1 mixture of PeriMag and KI (conc. 94 mg/ml). Thus, all of the letter ‘E’ contributes to MPI signal while only the letter ‘L’ is visualized by CT.

### Sequences

The CT system is set up to generate approx. one projection per second, which allows for an appropriate duty cycle for the MPI system to cool down. For 2D MPI and 3D CT data acquisition 100 projections covering 360° were acquired for sample 1 and 500 projections were acquired for sample 2. A trigger signal generated prior to each CT scan is used to run the sequence for the MPI system simultaneously. The CT projections were measured with an integration time of 200 ms each in a Stop-and-Go sequence where the sample was rotated a specific angle (360°/100 and 360°/500) and stopped before the acquisition of an individual projection. The beam was generated at 120 kV and filtered by 1 mm Al to remove beam hardening effects.

A trigger-out signal of MetRIC is used to run the MPI sequence using an arbitrary waveform generator (ArbStudio 1104D, Teledyne LeCroy, USA). It generates a sinusoidal signal with the frequency *f* = 10250 Hz featuring a linear ramping (ramp up as well as ramp down of about 4 ms) to avoid feedback issues from the audio amplifier and to avoid transient effects. The entire sequence has a total duration of about 30 ms. The central 20 ms are acquired with a digital oscilloscope (HDO8038A, Teledyne LeCroy, USA) with a sampling rate of 10 MS/s resulting in a data length of 200.000 data points. The receive chain consists of the gradiometric coils, a 20 kHz Chebychev high-pass filter (7-pole) and a 800 kHz butterworth low-pass filter (5-pole), both designed for 50 Ω.

The total scan time for *n* projections was set to *n* seconds with a duty cycle of 3.3% for MPI and 20% for CT measurement. This results in a total scan time of 100 seconds for sample 1 (100 projections) and 500 seconds for sample 2 (500 projections).

### Data Reconstruction

The data correction and filtering of the MPI data sets is performed using a custom reconstruction software^22^. In a preliminary measurement, the transfer function of the receive chain was determined using a small pick-up coil positioned in the center of the MPI system providing a signal sweep from 10 kHz to 1 MHz. All acquired MPI data is subtracted with an empty measurement to remove residual signals e.g. from the surrounding permanent magnets. After that, the amplitude and phase correction utilizing the transfer function is applied on the data. The corrected and filtered signal, which consists of 205 periods (20 ms·*f*_1_), is reduced to a half period^3–5^ representing the projected signal at the specific angle (one row of a sinogram). In a final step the sinogram rows were transferred to Matlab (The Mathworks, USA) and reconstructed using a standard filtered backprojection algorithm.

## Results

**Figure 9:**
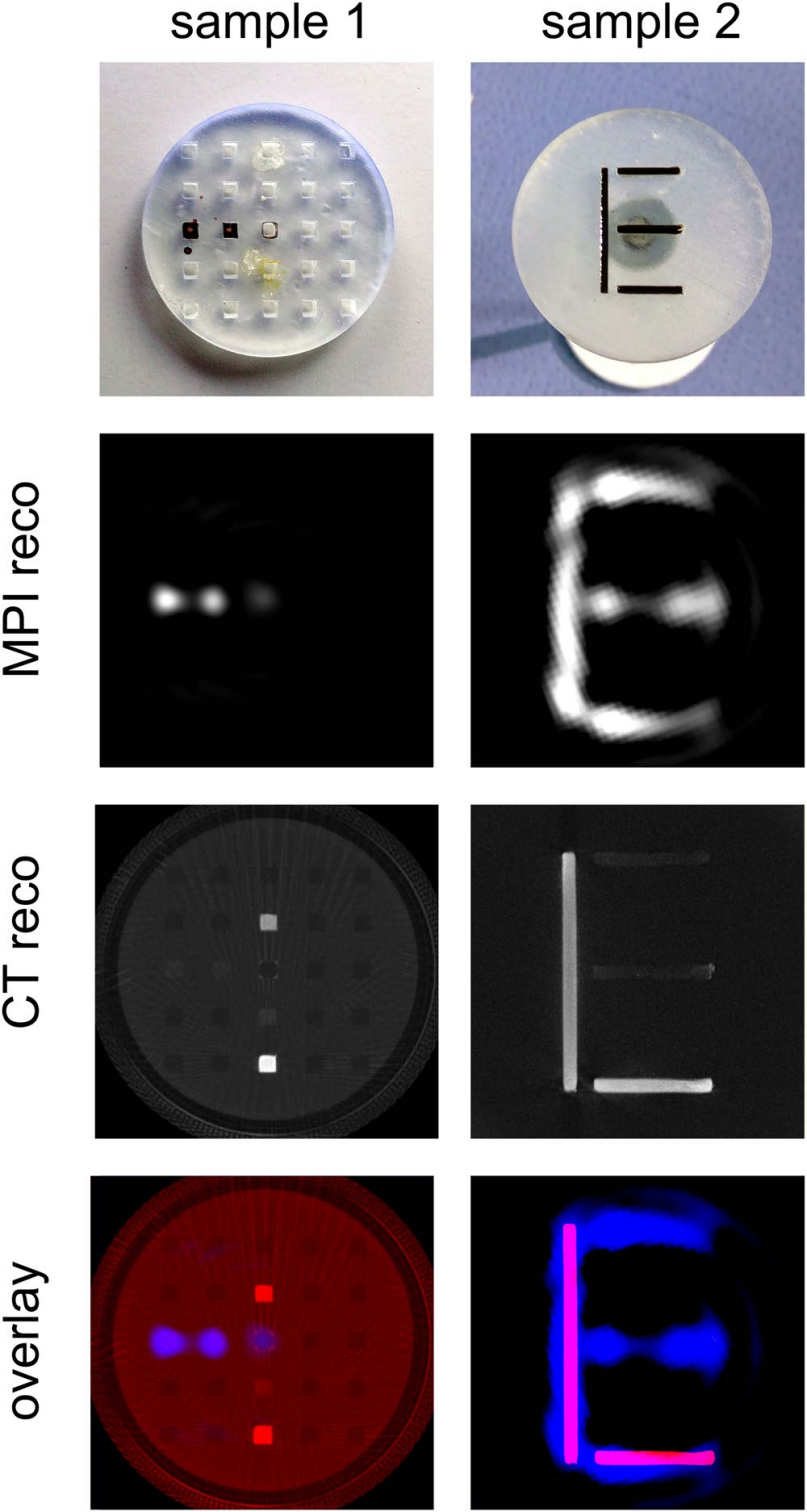
Results of simultaneous MPI CT measurement of sample 1 (**left**) and sample 2 (**right**).

The results of scanning both samples are shown in Fig. 9. The reconstruction of sample 1 (left column in Fig. 9) demonstrate the high transparency of PeriMag in the CT scan and the invisibility of potassium iodide in the MPI scan.

The reconstruction results of scanning sample 2 (right column in Fig. 9) show the reconstructed letter ‘E’ for EP5 in the MPI image and the reconstructed letter ‘L’ for LRM in the CT image.

## Discussion

In Fig. 9 (right – sample 2) the reconstructed CT image shows the complete letter ‘E’, but with extreme low contrast for the elements filled with PeriMag without potassium iodide. Even at high SPION concentration, the absorption at 120 kV is very weak. To visualize SPIONs directly with CT, the anode voltage has to be set to the absorption edge (K-edge) of Fe (6.4 kV). However, this is not an appropriate energy level for scanning large samples because of the high absorption of the material.

There is no direct influence or interference between both modalities, MPI and CT, such as stray magnetic fields which could perturb the X-ray anode’s focusing. This allows for truly simultaneous imaging, which is an enormous advantage compared to MPI-MRI hybrid scanners, that only allow sequential imaging due to incompatible magnetic field requirements^3–5^.

For MPI data reconstruction, a standard filtered backprojection algorithm is used. This algorithm does not take magnetic field inhomogeneities into account. One possible effect is the bending of the FFL at the edge of the FOV (see Supplementary Material S1). Another effect is the inhomogeneous magnetic field gradient along the FOV (see Supplementary Fig. S2). These effects can be avoided by reducing the size of the usable FOV to the homogeneous area or by using a system matrix approach for reconstruction, which takes into account magnetic field inhomogeneities and can remove the distortion as seen in Fig. 9 right^23^.

The resolution for MPI images strongly depends on the available gradient as well as the used tracer material^24^. For a gradient of about 4 T/m the expected resolution is on the order of 1 mm for Resovist® (Bayer, Germany) or similar tracers like Perimag® (Micromod, Germany).

For hybrid MPI-CT measurements using a rotating sample holder, the centering of the rotating axis of the sample inside the MPI system is difficult and requires more sophisticated approaches such as self-calibrating centering as described in detail by Dittmann *et al*.^25^.

The choice of permanent magnets instead of electromagnets for the generation of the strong gradient was firstly shown by Gleich and Weizenecker^1^. Several later MPI scanner uses electromagnets because of speediness. The presented Halbach system generates a static FFL in the center and offers similar encoding schemes as for CT. Furthermore, the Halbach system consumes less power and problems with electrical connections, arising from the system rotation, are avoided.

In a next generation, the Halbach magnet arrangement will be realized with three concentric rings around the measuring field^20^. This construction will allow the generation of magnetic field gradients of more than 5 T/m in a measuring field of 80 mm diameter with a usable FOV diameter of approximately 50 mm (2D). For 3D imaging, an additional mechanical movement of the gantry/sample is required.

## Conclusions and Outlook

As MPI is establishing itself as powerful tool in preclinical imaging, *in-vivo* applications become more important. Fast and simultaneous image acquisition may help to reduce inevitable motion artifacts caused by breathing and heartbeats. Retrospective coregistration is computationally intense, requires the use of fiducial markers and could be inaccurate due to slight motion during alternation of the imaging modality. Moreover, the benefits of simultaneous vs. sequential imaging in other hybrid modalities such as PET/CT have been broadly established^26–28^.

The first hybrid MPI-CT measurements are presented allowing simultaneous MPI and CT imaging of bimodal samples. A novel concept based on Halbach arrays is used for generating a static field free line featuring a strong magnetic field gradient. The newly developed MPI component offers an open design providing a CT ‘window’ for direct feedthrough of X-rays and simultaneous imaging. Hence, the SPION distribution (MPI) as well as the anatomical information of the surrounding material (CT) can be visualized simultaneously and rapidly. Combination of the highly sensitive tracer-based imaging method of MPI with the exact visualization of anatomical structures of CT can be a basis for improving diagnostic accuracy in preclinical imaging.

## Supplementary information

3D sketch of the MPI scanner and its field configuration

Simulation of magnetic gradient strength and magnetic field along the field of view (FOV) inside the MPI scanner
