# Supplementary material for: Magnetic Particle Imaging meets Computed Tomography: first simultaneous imaging
Source: Sci Rep. 2019 Sep 2;9:12627. doi: 10.1038/s41598-019-48960-1 (PMC6718383; doi:10.1038/s41598-019-48960-1)
Supplement: Supplementary file 1 — 3D sketch of the MPI scanner and its field configuration [file 41598_2019_48960_MOESM1_ESM.pdf]

Supplementary Information

Magnetic Particle Imaging meets Computed Tomography: first simultaneous imaging

Patrick Vogel [a,d,*], Jonathan Markert [a,c], Martin A. Rückert [a], Stefan Herz [d], Benedikt Keßler [c], Kilian Dremel [f], Daniel Althoff [f], Matthias Weber [e,+], Thorsten M. Buzug [e], Thorsten A. Bley [d], Walter H. Kullmann [c], Randolf Hanke [b,f], Simon Zabler [b,f], Volker C. Behr [a]

[a] Department of Experimental Physics 5 (Biophysics), University of Würzburg, 97074 Würzburg, Germany

[b] Department of Experimental Physics (X-Ray Microscopy), University of Würzburg, 97074 Würzburg, Germany

[c] Institute of Medical Engineering, University of Applied Sciences Würzburg-Schweinfurt, 97421 Schweinfurt, Germany

[d] Department of Diagnostic and Interventional Radiology, University Hospital Würzburg, 97080 Würzburg, Germany

[e] Institute of Medical Engineering, University of Lübeck, 23562 Lübeck, Germany

[f] Fraunhofer Development Center X-ray Technology EZRT, 97074 Würzburg, Germany

[+] now with Magnetic Insight Inc., Alameda CA, USA

* Corresponding author, email: Patrick.Vogel@physik.uni-wuerzburg.de

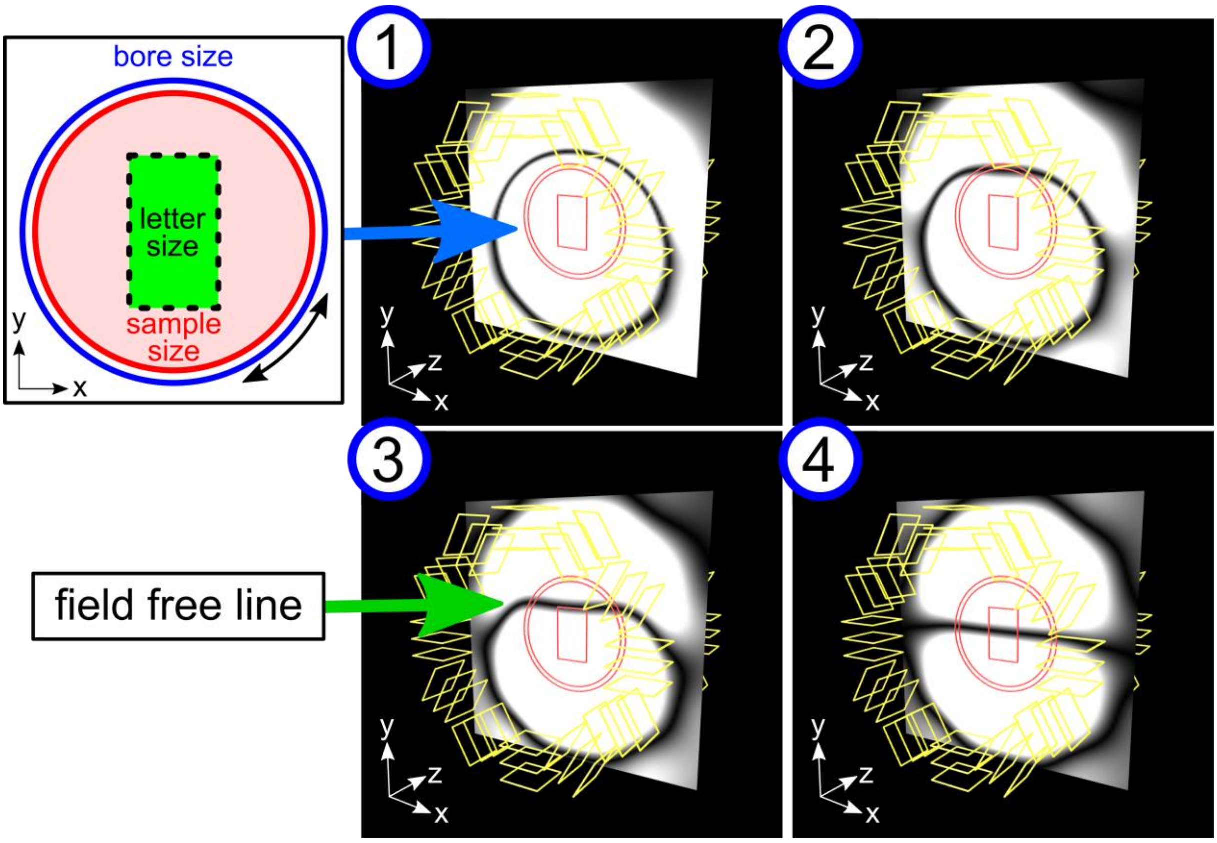

Supplementary Figure S1

3D sketch of the MPI scanner and its field configuration: The simulation of the MPI scanner shows the magnetic field distribution at different time points (1)-(4). The absolute value of the magnetic field is plotted to indicate regions with low magnetic field strength (dark areas). The yellow elements indicate the Halbach magnets, the blue ring the bore size of the MPI scanner and the red one the sample size. Inside the bore size (field of view – FOV) the field-free line (FFL) is only slightly bent. The sample is rotated around the z-axis to cover full projection data for reconstruction.
