# Supplementary material for: Magnetic Particle Imaging meets Computed Tomography: first simultaneous imaging
Source: Sci Rep. 2019 Sep 2;9:12627. doi: 10.1038/s41598-019-48960-1 (PMC6718383; doi:10.1038/s41598-019-48960-1)
Supplement: Supplementary file 2 — Simulation of magnetic gradient strength and magnetic field along the field of view (FOV) inside the MPI scanner [file 41598_2019_48960_MOESM2_ESM.pdf]

Supplementary Information

Magnetic Particle Imaging meets Computed Tomography: first simultaneous imaging

Patrick Vogel [a,d,*], Jonathan Markert [a,c], Martin A. Rückert [a], Stefan Herz [d], Benedikt Keßler [c], Kilian Dremel [f], Daniel Althoff [f], Matthias Weber [e,+], Thorsten M. Buzug [e], Thorsten A. Bley [d], Walter H. Kullmann [c], Randolf Hanke [b,f], Simon Zabler [b,f], Volker C. Behr [a]

[a] Department of Experimental Physics 5 (Biophysics), University of Würzburg, 97074 Würzburg, Germany

[b] Department of Experimental Physics (X-Ray Microscopy), University of Würzburg, 97074 Würzburg, Germany

[c] Institute of Medical Engineering, University of Applied Sciences Würzburg-Schweinfurt, 97421 Schweinfurt, Germany

[d] Department of Diagnostic and Interventional Radiology, University Hospital Würzburg, 97080 Würzburg, Germany

[e] Institute of Medical Engineering, University of Lübeck, 23562 Lübeck, Germany

[f] Fraunhofer Development Center X-ray Technology EZRT, 97074 Würzburg, Germany

[+] now with Magnetic Insight Inc., Alameda CA, USA

* Corresponding author, email: Patrick.Vogel@physik.uni-wuerzburg.de

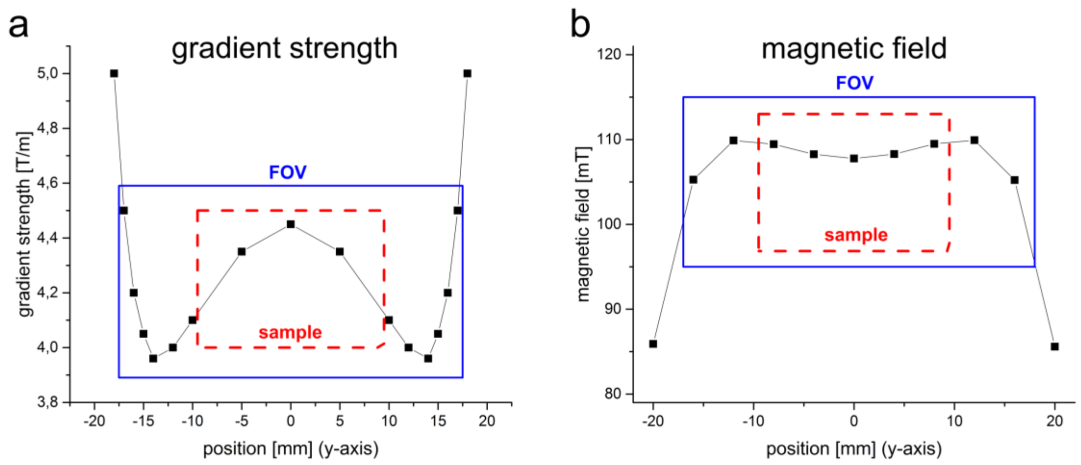

Supplementary Figure S2

Simulation of magnetic gradient strength and magnetic field along the field of view (FOV) inside the MPI scanner: a) the gradient strength varies about 12 percent in the FOV and 8 percent in the sample area. b) the magnetic field of the transmit coils (tx) varies about 2 percent within a radius of 15 mm.
